# Fast 3 T nigral hyperintensity magnetic resonance imaging in Parkinson’s disease

**DOI:** 10.1038/s41598-020-80836-7

**Published:** 2021-01-13

**Authors:** Gabriella Hernadi, David Pinter, Szilvia Anett Nagy, Gergely Orsi, Samuel Komoly, Jozsef Janszky, Norbert Kovacs, Gabor Perlaki

**Affiliations:** 1 Pecs Diagnostic Centre, Pecs, Hungary; 2 Department of Neurology, Medical School, University of Pecs, Pecs, Hungary; 3 MTA-PTE Clinical Neuroscience MR Research Group, Pecs, Hungary; 4 Neurobiology of Stress Research Group, Szentagothai Research Center, University of Pecs, Pecs, Hungary; 5 Department of Laboratory Medicine, Medical School, University of Pecs, Pecs, Hungary

**Keywords:** Parkinson's disease, Movement disorders

## Abstract

The absence of nigral hyperintensity is a promising MR marker for Parkinson’s disease (PD), but its small size imposes limitations on its routine use. Our aim was to compare Multi Echo Data Image Combination (MEDIC), segmented echo-planar imaging (EPISEG) and fluid-attenuated inversion recovery (FLAIR) sequences, as well as both magnitude (MAG) and susceptibility-weighted imaging (SWI) reconstructions of single-echo gradient echo for nigral hyperintensity imaging. Twenty-five healthy and twenty PD subjects were included. Sensitivity to motion artefacts, confidence of the radiologist in interpretation, rate of nondiagnostic scans and diagnostic accuracy were assessed. EPISEG was less motion-sensitive than MEDIC, MAG, and SWI, while FLAIR was less motion-sensitive than MAG and SWI. The reviewers were more confident when using EPISEG compared to any other techniques and MEDIC was superior to FLAIR. The proportions of nondiagnostic scans were lower for EPISEG than for other sequences. The best diagnostic performance was achieved for EPISEG (sensitivity = 65%, specificity = 96%). Using EPISEG, the absence of nigral hyperintensity in PD was associated with higher Hoehn-Yahr stage and MDS-UPDRS II + III. Nigral hyperintensity may be intact at the very early stages of PD. The promising properties of EPISEG may help the transfer of nigral hyperintensity imaging into daily clinical practice.

## Introduction

Parkinson’s disease (PD) is the second most common neurodegenerative disorder. At present, there are no 100% reliable in-vivo diagnostic markers for PD, thus the diagnosis can be established primarily based on the cardinal motor symptoms of the disease with the strong dependence on clinicians’ experience^1^.

Dopamine transporter single photon emission computed tomography (DaTSCAN) is useful for diagnostic purposes in PD^2^. However, DaTSCAN involves radiation exposure and it is more expensive and less widely available than MRI^3,4^. Besides nuclear medicine methods, a range of potential MRI signs of PD have been demonstrated^5^, and nigral hyperintensity is among the most promising ones. It consistently provides excellent diagnostic accuracy^6^.

Using in-vivo 7T T2*-weighted MRI, Kwon et al.^7^ observed a relatively higher signal region in the lateral portion of the substantia nigra in normal controls, whereas this region (i. e. nigral hyperintensity) was not visible in subjects with PD. Later, by combining 7T MRI and histologic data, Blazejewska et al.^8^ found that the oval hyperintense region conforms to nigrosome 1 subregion of substantia nigra, showing particularly severe loss of dopamine-containing neurons in PD. Since these pioneering ultra-high field studies, the normal appearance of nigral hyperintensity and its loss in PD have also been replicated at 3T field-strength, which is more widely available in clinical practice^3,6,9–13^. However, given the small size of the detectable hyperintensity, high-resolution susceptibility-weighted MRI with adequate contrast-to-noise/signal-to-noise ratio is required^3,14^. Due to the limitations of a 3T clinical scanner compared to a 7T scanner, nigral hyperintensity at 3T is usually imaged with relatively long (5:45–9:57) acquisition time^8,11,14–18^ or with low slice resolution (≥ 2 mm)^9,13,19–24^, which was found to be suboptimal^25^. To address this challenge, multi-echo sequences or multi-shot echo-planar imaging (EPI) acquisition techniques were used by some groups^3,10,12,26^.

To the best of our knowledge, no previous studies have compared these two techniques for nigral hyperintensity detection using the same subjects. Our overall goal was to compare Multi Echo Data Image Combination (MEDIC) and phase-segmented EPI (EPISEG) sequences acquired with the same resolution parameters to determine which is more eligible for nigral hyperintensity imaging at 3T. The measurement was aimed to be quick (< 5 min) and manufacturer-available pulse sequences requiring no sequence programming or any extra offline postprocessing were used to increase the potential applicability in everyday clinical practice. Resolution was aimed to be similar to that recently suggested to be high enough to consistently visualize nigral hyperintensity (~ 18% smaller voxel size in our case)^27^.

Because some of the previous studies were based on routine susceptibility-weighted imaging (SWI)^9,22,28^, we additionally tested the performance of both the magnitude (MAG) and SWI reconstructions of a whole-brain routine clinical single-echo fast low angle shot (FLASH) sequence unoptimized (e.g. resolution) for nigral hyperintensity. There is only a single study suggesting that nigral hyperintensity can be visualized on 3D fluid-attenuated inversion recovery (FLAIR) images, therefore this sequence was also tested using the resolution reported previously^29^.

## Methods

### Subjects

Twenty-five healthy subjects (10 men; mean age: 63.3 ± 8.0, range: 43–73 years) and twenty PD patients (9 men; mean age: 59.7 ± 11.4, range: 42–77 years) were included. Healthy subjects were recruited through personal contacts of the authors. None of them reported symptoms of rapid eye movement sleep behavior disorder (RBD) or hyposmia. The median probability of prodromal PD was 0.4% (range: 0.05–4.66%) as calculated using the Movement Disorder Society (MDS) research criteria based on the following self-reported factors^30^: age, sex, regular pesticide exposure, occupational solvent exposure, nonuse of caffeine, smoking, family history of PD or known genetic mutation, olfactory loss, constipation, excessive daytime somnolence, symptomatic hypotension, urinary dysfunction, diagnosis of depression.

PD was diagnosed based on the UK Parkinson’s Disease Society Brain Bank Diagnostic Criteria and patients with previous abnormal DaTSCAN imaging were recruited. The only patient without DaTSCAN examination had Hoehn-Yahr stage 2 and disease duration of 5 years demonstrating good levodopa-response. To assess disease severity, the composite Part II + III score of the MDS-sponsored Unified Parkinson's Disease Rating Scale (MDS-UPDRS II + III)^31,32^, the Hoehn–Yahr (H&Y) stage and disease duration were used. Demographic and clinical data are presented in Supplementary Table S1. For making our results comparable with those of previous studies, separate MDS-UPDRS Part II and Part III data were also reported and converted to UPDRS Part II and Part III scores using the method suggested by Goetz et al. (Supplementary Table S1)^33^. All subjects got detailed information on the investigation and informed consent was obtained from all participants. The study was approved by Institutional and Regional Ethical Board of the University of Pécs (7069-PTE 2018) and was performed in accordance with the ethical standards of the 1964 Declaration of Helsinki and its later amendments.

### Magnetic resonance imaging

All subjects were scanned on the same 3T MRI scanner (MAGNETOM Prisma^fit^, Siemens Healthcare, Erlangen, Germany) with a 20-channel Head/Neck coil.

Three-dimensional sagittal *FLAIR* image was acquired for each subject (TR/TI/TE = 5000/1800/388 ms; 320 sagittal slices with nominal slice thickness = 0.5 mm; slice resolution = 50%; FOV = 256 × 256 mm^2^; matrix = 256 × 256 reconstructed to 512 × 512; turbo factor = 278; receiver bandwidth = 751 Hz/pixel; GRAPPA acceleration factor = 2; acquisition time = 5:57).

Three-dimensional *EPISEG* (“ep_seg_fid”) measurement was also obtained (TR/TE = 150/36 ms; Flip Angle = 30; 28 axial slices; slice thickness = 1 mm; FOV = 180 × 180 mm^2^; matrix = 256 × 256 reconstructed to 512 × 512; receiver bandwidth = 888 Hz/pixel; 6 averages; EPI factor = 53; fat suppression = on; acquisition time = 2:08).

Three-dimensional *MEDIC* gradient echo sequence, combining the images of individual echoes into a single image, was acquired with 6 echoes (TR/Te_eff_ = 64/35 ms; Flip Angle = 22°; 26 axial slices; slice thickness = 1 mm; FOV = 180 × 180 mm^2^; matrix = 256 × 256 reconstructed to 512 × 512; receiver bandwidth = 120 Hz/pixel; GRAPPA acceleration factor = 2; elliptical scanning = on; acquisition time = 4:19).

Whole-brain routine clinical three-dimensional FLASH sequence unoptimized for nigral hyperintensity visualization was also performed and both the *MAG* and *SWI* (i.e. combined magnitude and phase information) images were automatically reconstructed by the scanner (TR/TE = 27/20 ms; Flip Angle = 15°; 88 axial slices; slice thickness = 1.5 mm; FOV = 230 × 172.5 mm^2^; matrix size = 256 × 182 reconstructed to 256 × 192; receiver bandwidth = 120 Hz/pixel; GRAPPA acceleration factor = 2; acquisition time = 4:51).

In order to ensure consistence and to minimize undesirable magnetic susceptibility effects at air/tissue and bone/tissue interfaces, axial slices were acquired parallel to the base of the skull with exactly the same angulation (using the copy reference option of the scanner) for all of the above axial sequences^26^.

Main scan parameters for the above sequences are also summarized in Supplementary Table S2.

### Visual evaluation

Anonymized MR images without any clinical data were visually evaluated in randomized order, in consensus by a neuroradiologist (G.H with 7 years of experience in reporting brain MRI) and a postdoctoral researcher (G.P. with 10 years of experience in processing brain MRI). The images were simultaneously reviewed in axial and orthogonal coronal planes as they were acquired (i.e. without reformatting the image). Reviewers were allowed to view the images in reformatted axial plane perpendicular to cerebral aqueduct and its orthogonal coronal plane as well, if it was necessary.

The evaluation was performed with 3DSlicer 4.10.2 (r28257) using a 24″ monitor with 5th generation AMVA panel calibrated to 120 cd/m^2^, 6500 K, and gamma of 2.2 (resulting in contrast ratio over 3000:1).

First, all scans were scored for movement-related artefacts on a 3-point ordinal scale (0 = little/no artefact; 1 = moderate artefact; 2 = excessive artefact). Scans with excessive motion artefacts were rated as *nondiagnostic* and excluded from further evaluation.

The visibility of nigral hyperintensity was separately rated for each hemisphere on a 3-point ordinal scale (0 = not visible; 1 = probably present; 2 = clearly present). The confidence of the reviewers in their interpretation (i.e. how reliable the presence/absence of nigral hyperintensity could be assessed) was also scored separately for each hemisphere on a 3-point ordinal scale (0 = low confidence; 1 = moderate confidence; 2 = high confidence). Scans with low confidence at both hemispheres were rated as *nondiagnostic*. Taking into consideration the asymmetrical onset of PD, scans with nigral hyperintensity probably/clearly present at one hemisphere with moderate/high confidence, but low confidence at the other hemisphere were also rated as *nondiagnostic*.

The scans were classified as *abnormal* if nigral hyperintensity was at least unilaterally not visible, *normal* if hyperintensity was clearly present bilaterally or clearly present unilaterally and probably present contralaterally, and *nondiagnostic* if hyperintensity was probably present bilaterally^3^.

### Statistical analysis

Statistical analyses were performed using IBM SPSS Statistics for Windows, Version 23.0 (IBM Corp., Armonk, NY, USA). Since the score for confidence of nigral hyperintensity assessment was not different between the hemispheres (*P* > 0.05, as assessed by Sign test) in either controls or patients for any of the examined sequences, confidence level was used as the average of left- and right confidence levels in all further statistics.

Sex distribution was compared between patients and controls using Fisher’s exact test, while age and education years were compared by Mann–Whitney U-test.

The distributions of nondiagnostic scans and movement-related artefacts were compared between patients and controls using Fisher's exact test, separately for each sequence. Confidence level was compared between patients and controls using Mann–Whitney U-test. To account for multiple comparisons, Benjamini–Hochberg correction was applied with q = 0.05 and a total number of comparisons of 5 (i.e. 5 MRI sequences).

The difference between sequences in the proportions of nondiagnostic scans was assessed from all subjects using McNemar’s test. Differences between sequences in movement-related artefacts and confidence levels were assessed from all available subjects using Sign test, separately for each pair of sequences (e.g. MEDIC vs. EPISEG). To account for multiple comparisons, Benjamini–Hochberg correction was applied with q = 0.05 and a total number of comparisons of 10 (i.e. 10 possible pairing of the sequences).

To compare the diagnostic accuracy across sequences, receiver operating characteristic (ROC) analysis with clinical diagnosis (PD vs. control) as reference standard was run for each sequence. Area under the ROC curve (AUC), sensitivity, and specificity were calculated. Nondiagnostic scans were excluded from these analyses.

It was also assessed whether nigral hyperintensity-based normal/abnormal classification of PD patients are related to the severity of PD, age or sex. Fisher’s exact test was performed to compare the proportions of normal and abnormal classifications between H&Y1 and H&Y2 subgroups as well as between males and females. Mann–Whitney U-test was used to test differences in MDS-UPDRS II + III, disease duration, and age between patients with normal and abnormal nigral hyperintensity appearance. In order to control for the potential effects of antiparkinsonian pharmacotherapy on MDS-UPDRS II + III, MDS-UPDRS II + III was also compared between the two patient subgroups by multiple linear regression analysis including levodopa equivalent daily dose (LEDD)^34^ as covariate; LEDD was square root transformed to reduce skewness. These analyses were performed only for the EPISEG sequence, because the nondiagnostic scans reduced the already small number of patients available for analysis regarding other sequences. To account for multiple comparisons, Benjamini–Hochberg correction was applied with q = 0.05 and a total number of comparisons of 5 (i.e. 5 variables examined).

Uncorrected P-values are reported to facilitate comparisons to other studies, but *P* values surviving correction for multiple comparisons are highlighted in bold and considered as significant findings.

## Results

Sex distribution, age and education years were not significantly different between patients and controls (Supplementary Table S1). Confidence level in interpretation and the distributions of nondiagnostic scans or movement-related artefacts were not significantly different between patients and controls for any of the sequences (Tables 1, 2).

**Table 1:** Comparison of the distributions of nondiagnostic scans and motion-artefact corrupted images between patients and controls for each MRI sequence.

MR sequences	PD (n = 20)	Control (n = 25)	*P* value^a^
**Nondiagnostic scans**			
EPISEG	0 (0%)	0 (0%)	n.a
FLAIR	8 (40%)	8 (32%)	0.755
MEDIC	2 (10%)	6 (24%)	0.269
MAG	8 (40%)	8 (32%)	0.755
SWI	6 (30%)	6 (24%)	0.741
**Movement artefacts**			
EPISEG	19/1/0 (5%)	23/2/0 (8%)	> 0.999
FLAIR	16/1/3 (20%)	21/4/0 (16%)	0.122
MEDIC	13/7/0 (35%)	14/9/2 (44%)	0.667
MAG	12/5/3 (40%)	12/9/4 (52%)	0.778
SWI	11/4/5 (45%)	12/10/3 (52%)	0.287

Data are presented as total number (%) of subjects with nondiagnostic scans and number of subjects with no/moderate/excessive movement artefacts (% of subjects with moderate or excessive movement artefacts).

*PD* Parkinson’s disease, *EPISEG* 3D segmented echo-planar imaging, *FLAIR* 3D fluid-attenuated inversion recovery, *MEDIC* 3D multi echo data image combination gradient echo, *MAG* magnitude reconstruction of 3D single-echo fast low angle shot gradient echo, *SWI* SWI reconstruction of 3D single-echo fast low angle shot gradient echo, *n.a.* not applicable.

^a^Fisher’s exact test (2-sided exact *P* value). None of the uncorrected *P* values survive Benjamini–Hochberg correction for multiple comparisons calculated using q = 0.05 and a total number of comparisons of 5.

**Table 2:** Comparison between patients and controls regarding the confidence of reviewers in nigral hyperintensity assessment.

MR sequences	Group	Number^a^	Mean rank	*P* value^b^
EPISEG	PD	20	21.13	0.080
	Control	25	24.50	
FLAIR	PD	17	21.5	> 0.999
	Control	25	21.5	
MEDIC	PD	20	24.93	0.078
	Control	23	19.46	
MAG	PD	17	20.79	0.479
	Control	21	18.45	
SWI	PD	15	15.13	0.026
	Control	22	21.64	

*PD* Parkinson’s disease, *EPISEG* 3D segmented echo-planar imaging, *FLAIR* 3D fluid-attenuated inversion recovery, *MEDIC* 3D multi echo data image combination gradient echo, *MAG* magnitude reconstruction of 3D single-echo fast low angle shot gradient echo, *SWI* SWI reconstruction of 3D single-echo fast low angle shot gradient echo.

^a^The number of available subjects after exclusion due to excessive motion artefacts.

^b^Mann-Whitney U-test (2-sided exact *P* value). None of the uncorrected *P* values survive Benjamini–Hochberg correction for multiple comparisons calculated using q = 0.05 and a total number of comparisons of 5.

EPISEG was significantly less sensitive to motion compared to MEDIC, MAG, and SWI techniques. FLAIR was less sensitive to motion compared to the MAG and SWI (Table 3).

**Table 3:** Pairwise comparison between MRI sequences.

MR sequences	FLAIR	MEDIC	MAG	SWI
**Movement artefact**^a^				
EPISEG	1/6/38 *P* = 0.125	**1/17/27 P < 0.001**^**c**^	**1/20/24 P < 0.001**^**c**^	**1/20/24 P < 0.001**^**c**^
FLAIR		5/12/28 *P* = 0.143	**2/15/28 P = 0.002**^**c**^	**1/16/28 P < 0.001**^**c**^
MEDIC			4/11/30 *P* = 0.118	5/14/26 *P* = 0.064
MAG				3/5/37 *P* = 0.727
**Confidence**^a^				
EPISEG	**23/1/18 P < 0.001**^**c**^	**11/2/30 P = 0.022**^**c**^	**16/2/20 P = 0.001**^**c**^	**11/2/24 P = 0.022**^**c**^
FLAIR		**6/20/14 P = 0.009**^**c**^	7/11/19 *P* = 0.481	4/14/19 *P* = 0.031
MEDIC			14/5/19 *P* = 0.064	9/8/19 *P* > 0.999
MAG				6/11/19 *P* = 0.332
**Nondiagnostic scan**^b^				
EPISEG	**0/16 P < 0.001**^**c**^	**0/8 P = 0.008**^**c**^	**0/16 P < 0.001**^**c**^	**0/12 P < 0.001**^**c**^
FLAIR		12/4 *P* = 0.077	7/7 *P* = 1.000	8/4 *P* = 0.388
MEDIC			2/10 *P* = 0.039	4/8 *P* = 0.388
MAG				7/3 *P* = 0.344

*Confidence* Confidence of reviewers in interpretation, *EPISEG* 3D segmented echo-planar imaging, *FLAIR* 3D fluid-attenuated inversion recovery, *MEDIC* 3D multi echo data image combination gradient echo, *MAG* magnitude reconstruction of 3D single-echo fast low angle shot gradient echo, *SWI* SWI reconstruction of 3D single-echo fast low angle shot gradient echo.

^a^Values are presented as the number of cases where the sequence in *row* achieved *higher/lower/equal* score compared to the sequence in *column*. 2-sided exact *P* values are based on Sign test.

^b^Values are presented as the number of discordant pairs where only the sequence in *row/column* is nondiagnostic, while the other is diagnostic. 2-sided exact *P* values are based on McNemar’s test.

^c^These uncorrected *P* values in bold survive Benjamini–Hochberg correction for multiple comparisons calculated using q = 0.05 and a total number of comparisons of 10.

The reviewers were more confident in assessing nigral hyperintensity using EPISEG compared to any of the other sequences, and they were more confident when using MEDIC compared to FLAIR. The proportions of nondiagnostic scans were significantly lower for EPISEG compared to any of the other sequences (Table 3).

**Figure 1:**
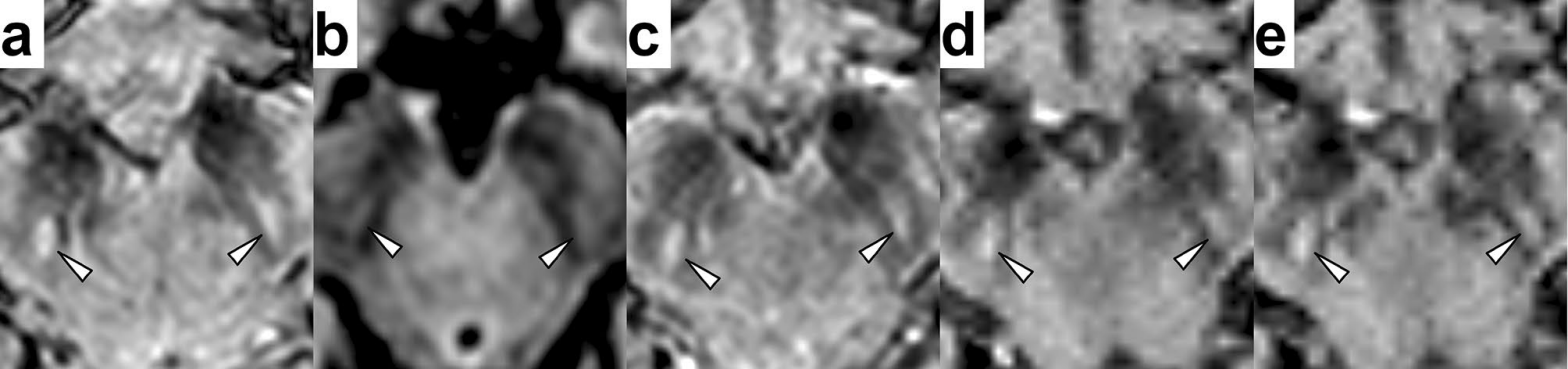
True-negative interpretation of the images of a 63-year-old healthy female subject. Nigral hyperintensity was bilaterally rated as “clearly present” (white arrowheads) on EPISEG (**a**), FLAIR (**b**), MEDIC (**c**), MAG (**d**), and SWI (**e**) images as well. Images are shown in radiological convention (right = subject’s left) at a level below the red nucleus.

**Figure 2:**
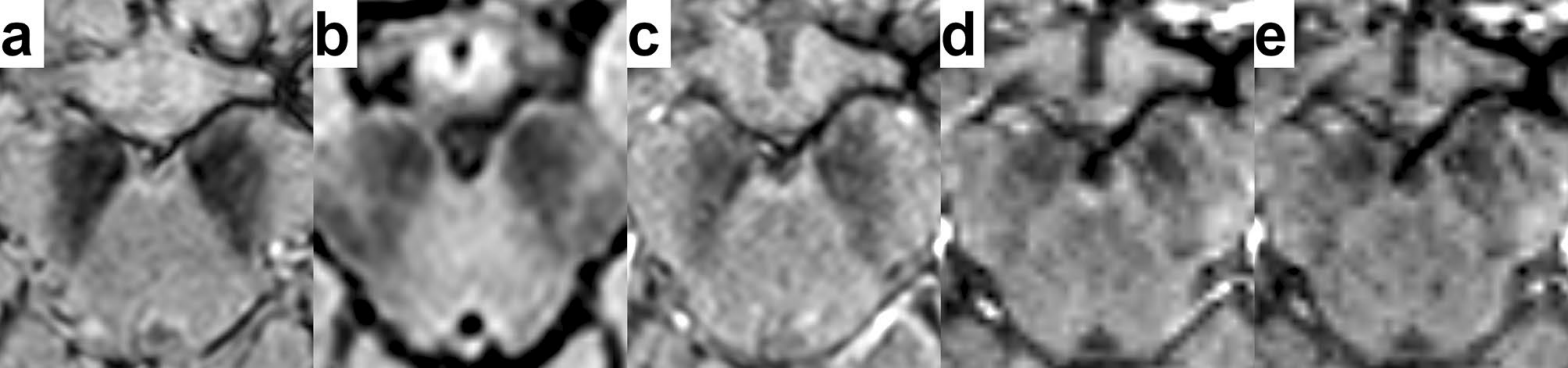
True-positive interpretation of the images of a 62-year-old woman with PD (Hoehn-Yahr stage: 2, MDS-UPDRS Part II: 6 points, MDS-UPDRS Part III: 24 points). Nigral hyperintensity was bilaterally rated as “not visible” on EPISEG (**a**), FLAIR (**b**), MEDIC (**c**), MAG (**d**), and SWI (**e**) images as well. Images are shown in radiological convention (right = subject’s left) at a level below the red nucleus.

**Figure 3:**
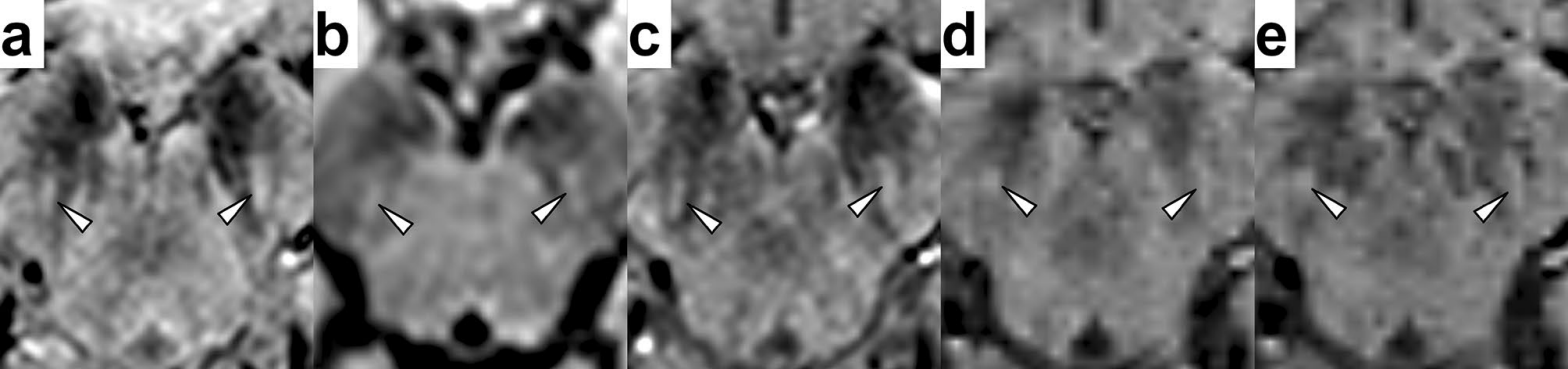
False-negative interpretation of the images of a 45-year-old woman with PD (Hoehn-Yahr stage: 1, MDS-UPDRS Part II: 2 points, MDS-UPDRS Part III: 4 points). Nigral hyperintensity was bilaterally rated as “clearly present” (white arrowheads) on EPISEG (**a**), FLAIR (**b**), MEDIC (**c**), MAG (**d**), and SWI (**e**) images as well. Images are shown in radiological convention (right = subject’s left) at a level below the red nucleus.

The diagnostic accuracy of each method is presented in Table 4. Examples of true-negative, true-positive, and false-negative readings are presented respectively in Figs. 1, 2 and 3. The best AUC (= 0.805) was achieved for the EPISEG sequence with sensitivity of 65% and specificity of 96%.

**Table 4:** Diagnostic accuracy of the different sequences.

MR sequence	N_patients_	N_controls_	AUC	Sensitivity (%)	Specificity (%)
EPISEG	20	25	0.805	65	96
FLAIR	12	17	0.750	50	100
MEDIC	18	19	0.753	61.1	89.5
MAG	12	17	0.762	58.3	94.1
SWI	14	19	0.714	42.9	100

Sensitivity and specificity are based on the diagnostic scans only.

*N*_*patients*_* and N*_*controls*_ number of diagnostic scans in patients and controls, respectively, *AUC* area under the ROC curve, *EPISEG* 3D segmented echo-planar imaging, *FLAIR* 3D fluid-attenuated inversion recovery, *MEDIC* 3D multi echo data image combination gradient echo, *MAG* magnitude reconstruction of 3D single-echo fast low angle shot gradient echo, *SWI* SWI reconstruction of 3D single-echo fast low angle shot gradient echo.

The normal/abnormal classification of PD patients based on nigral-hyperintensity assessment with EPISEG was related to disease severity (Table 5). The abnormal group showed significantly higher MDS-UPDRS II + III composite score and includes more patients with H&Y stage 2 (76.9% versus 14.3%). Disease duration, age, and sex showed no significant effect on this classification.

**Table 5:** Comparison between PD patients with normal and abnormal nigral hyperintensity appearance on EPISEG images.

Nigral hyperintensity		*P* value
Abnormal (n = 13)	Normal (n = 7)	
**MDS-UPDRS II + III**		
21.5 ± 9.0 [2–32]^a^	9.0 ± 3.8 [4–15]	**0.004**^b,e^ **0.005**^d,e^
**DD (years)**		
4.0 ± 2.4 [1–9]	3.1 ± 1.2 [2–5]	0.466^b^
**H&Y**		
Stage 1: 3 cases	Stage 1: 6 cases	**0.017**^c,e^
Stage 2: 10 cases	Stage 2: 1 case	
**Age (years)**		
60.7 ± 11.1 [42–73]	57.8 ± 12.6 [45–77]	0.588^b^
**Sex (M/F)**		
8/5	1/6	0.070^c^

Data are presented as mean ± standard deviation [range].

*PD* Parkinson’s disease, *EPISEG* 3D segmented echo-planar imaging, *MDS-UPDRS* Movement Disorder Society-sponsored Unified Parkinson’s Disease Rating Scale, *DD* disease duration, *H&Y* Hoehn–Yahr stage, *M* male, *F *female.

^a^Two patients were excluded (one for missing Part II and one for missing Part III score).

^b^Mann–Whitney U-test (2-sided exact *P* value).

^c^Fisher’s exact test (2-sided exact *P* value).

^d^Multiple linear regression analysis including levodopa equivalent daily dose as covariate.

^e^These uncorrected *P* values in bold survive Benjamini–Hochberg correction for multiple comparisons calculated using q = 0.05 and a total number of comparisons of 5.

## Discussion

In the current study, different manufacturer-available 3D MR sequences were compared for the evaluation of nigral hyperintensity. Our main goal was to compare MEDIC and EPISEG sequences acquired with the same resolution parameters. Single-echo FLASH (i.e. MAG, SWI) and FLAIR sequences were also tested with the resolution used in a typical routine clinical MRI protocol or suggested by the literature^29^, respectively.

Patient motion resulting in MR image degradation is associated with substantial extra costs^35^. Both the MAG and the SWI reconstructions of the single-echo FLASH sequence were found to be more sensitive to movement artefacts than FLAIR. The vulnerability of SWI to motion artefacts is known^36^ and has also been reported by other studies on nigral hyperintensity^9,37^. EPISEG was rated as less sensitive to motion compared to MEDIC, MAG, and SWI techniques. The relative motion insensitivity of EPISEG suggests that this sequence may provide a practical solution when nigral hyperintensity should be assessed in patients with involuntary movements and no special motion correction methods are available on the scanner for T2* acquisitions. In addition, if motion artefacts are excessive, the speed of EPISEG (~ 2 min) may permit repeating the measurement in the same session after prompting the patient, which is likely to decrease the rate of nondiagnostic scans^3^.

The confidence in expressing diagnostic judgment based on nigral hyperintensity was previously shown to be dependent on magnetic field strength (i.e. 3T vs. 7T)^12^. Our results indicated that it is also highly dependent on the imaging protocol used. The confidence of the radiologist and the rate of nondiagnostic scans are both of great importance to the referring physician in further decision making. FLAIR was inferior to both MEDIC and EPISEG regarding confidence, which might be related to the different contrast mechanism of FLAIR^29^. The highest confidence was achieved for the EPISEG. Furthermore, using this sequence, all scans were diagnostic, while the other sequences provided nondiagnostic images in 17.8–35.6% of the 45 subjects.

The EPISEG showed the best diagnostic performance with an AUC = 0.805, that is considered to be in the lower part of the excellent range (0.8–0.9)^38^. The other techniques had somewhat worse performance (AUC = 0.714–0.750) and provided AUC in the lower half of the acceptable range (0.7–0.8). However, in case of EPISEG all subjects could be included in the ROC analysis, while for the other four techniques the scans of several subjects (17.8–35.6%) were rated as undiagnostic and had to be excluded from the analysis. This may hinder direct comparison between the sequences in this sense. Forcing the reviewers to make decisions (i.e. normal or abnormal) based on these undiagnostic scans may reduce the diagnostic accuracy, as it has been demonstrated by previous studies when also including non-diagnostic scans (‘intent to diagnose’)^3,9^.

Using EPISEG, a relatively large number (n = 7) of false-negative cases (i.e. nigral hyperintensity bilaterally present in PD) were observed. Six of these seven patients were also interpreted as normal based on MEDIC, and the other techniques also produced false-negative findings in all cases if the images were diagnostic (5, 4 and 6 cases for the FLAIR, MAG and SWI techniques, respectively). This suggests that false-negativity of these patients is rather related to our sample and not sequence-specific. Using EPISEG, age and sex showed no significant association with normal/abnormal classification of PD patients. Moreover, controls were classified as normal in 24/25 cases irrespective of age and sex, supporting previous findings that aging-related iron accumulation and sex probably do not affect the visibility of nigral hyperintensity^22^. Disease duration was not different between false-negative and true-positive patient groups, which is in line with a previous study^20^. This finding is also supported by the non-significant correlation of disease duration with T2*-weighted signal in any of the nigrosomes^39^. However, we found a relationship between normal/abnormal classification of PD patients and disease severity measured by H&Y stage and composite MDS-UPDRS II + III score, which suggests that nigral hyperintensity may be intact at the very early stages of PD. In our case, the MDS-UPDRS II + III score of PD patients having normal nigral hyperintensity was significantly lower than that of patients with abnormal nigral hyperintensity (9.0 ± 3.8 vs. 21.5 ± 9.0, *P* = 0.004) and this magnitude of difference is clinically relevant by exceeding the minimal clinically important thresholds^31,40,41^. Somewhat conflicting with this interpretation, De Marzi et al.^22^ demonstrated the loss of nigral hyperintensity in at least two thirds of patients with idiopathic rapid eye movement sleep behavior disorder (iRBD), a disease representing a prodromal marker of neurodegenerative synucleinopathies, including PD. Bae et al.^42^ demonstrated that iRBD cases with nigral hyperintensity loss also showed reduced ^123^I-FP-CIT binding, while in other iRBD cases with intact ^123^I-FP-CIT binding, nigral hyperintensity was also intact. On the other hand, a negative association between the severity of PD and T2*-weighted signal in nigrosome 1 was also demonstrated^39^, which may support our findings.

Sensitivity of nigral hyperintensity loss for the diagnosis of PD ranged from 71 to 100% in previous 3T MRI studies^3,9,10,12,20^. In the present study, the relatively lower sensitivity (i.e. 65% for EPISEG) may be attributable to the study population representing relatively early stages of PD. All of our patients were in H&Y stage ≤ 2 and considering the cut-off points by Martínez-Martín et al.^43^, all of them fell into the mild category based on MDS-UPDRS Part III (≤ 32 points). Regarding MDS-UPDRS Part II scores, with the exemption of two subjects who had moderately severe PD (13 points ≤ and ≤ 29 points), all patients represented the category of mild disease (≤ 12 points).

The comparison of our sensitivity values to those of earlier studies is not easy and should be carefully considered because each study used its own imaging protocol, excluded different number of subjects based on image quality and investigated patients with varying disease severity. Analyzing only the images of patients with H&Y stage 2 in our study, the sensitivity of EPISEG increased to 91% (i.e. only 1 false-positive per 11 patients, which is within the range of literature values). This suggests that sensitivity values given by studies including greater proportions of patients with H&Y ≥ 2 may not be representative for earlier stages of PD.

Previous studies have usually not specified the exact proportions of H&Y1 and H&Y2 patients^3,9,12,15,18,21,23,26,37,44,45^, but most of them reported higher mean H&Y score than our one^3,9,15,18,23,37,44,45^, which may increase the sensitivity. Others included a relatively lower number of H&Y1 PD cases^10,11,46^, which may hinder the comparison with our study. Most of the previous studies reported higher scores (i.e. mean, median, minimum or lower and upper quartile) for MDS-UPDRS Part III^37^ or UPDRS Part III and/or UPDRS Part II^9–12,16,17,20,22,23,44,46^ than the corresponding scores of our patients, which may also increase the sensitivity. Sung et al.^16^ included 89 early-stage (H&Y1 and H&Y2) PD patients without false-negative interpretation, but the median, the lower and the upper quartiles of their UPDRS II and III scores were all higher than in our patients. Bae et al. also included several early-stage PD patients (H&Y1: 57 and H&Y2: 53). Despite the higher mean UPDRS III motor score of their patients, they still reported PD cases showing intact bilateral nigral hyperintensity, while demonstrating nigrostriatal degeneration on ^123^I-FP-CIT SPECT^20^, which suggests that nigrostriatal functional changes may develop earlier than structural changes indicated by the absence of nigral hyperintensity^10^. However, the discrepancy between the two techniques needs to be further investigated.

Despite the limited sensitivity, abnormal appearance of nigral hyperintensity still has diagnostic utility because it may reinforce clinical diagnosis with high specificity. In our study, the specificity of all five techniques (89.5–100%) was comparable to that of previous 3T studies (83.6–100%)^3,10–12,20^. The only healthy control with bilateral abnormal nigral hyperintensity appearance on EPISEG was rated as bilaterally abnormal using MEDIC as well, while the other three techniques were undiagnostic due to low confidence. It cannot be completely excluded that this subject has presymptomatic parkinsonism, especially because DaTSCAN examination was not an inclusion criterion for our control group.

Our goal was to compare manufacturer-available techniques that allow the interpretation right after scanning. Recently, a new MR imaging approach (referred to as susceptibility map-weighted imaging or true SWI) was proposed to assess nigral hyperintensity^14,27^, but this technique requires extra offline postprocessing and is therefore not included in the present comparison. However, this approach is not perfect either, given more than 20% of patients with PD showed bilateral nigral hyperintensity in a recent study^27^. To further improve the compatibility with the clinical environment, we used a 20-channel Head/Neck coil that is widely available in a clinical setting.

### Limitations

Our study has some limitations. First, the number of subjects is relatively low. Further sequence comparison studies with more participants are needed to find optimal nigral hyperintensity imaging technique at 3T. In addition, only early-stage PD patients were recruited which may introduce a selection bias. The clinical diagnosis used as reference may be imperfect due to the lack of postmortem confirmation. To minimize the possibility of any misdiagnosis, all of our PD patients were diagnosed by the same neurologist specialized in movement disorders and patients having abnormal DaTSCAN imaging were recruited. FLAIR, MAG, and SWI were acquired with relatively lower resolution. However, our goal was the comparison of EPISEG and MEDIC with whole-brain MAG/SWI optimized for routine clinical application rather than nigral hyperintensity assessment and with whole-brain FLAIR acquired with the same resolution as reported previously^29^. Undiagnostic scans may inflate AUC, sensitivity and specificity values for the MEDIC, FLAIR, MAG, and SWI techniques^3^. The availability and the precise implementations of the techniques compared in this study may vary among the major MR vendors. Since there were only a few patients in whom nigral hyperintensity was unilaterally lost (i.e. ≤ 3 cases for each technique), the relationship between clinical asymmetry and the lateralization of nigral hyperintensity loss could not be assessed in the present study. The main strengths of our study include comparing different MRI sequences on the same subjects, including patients with relatively early stages of PD, and assessing disease severity dependence of the visibility of nigral hyperintensity.

## Conclusions

In conclusion, tailored MRI protocols are important for nigral hyperintensity imaging. EPISEG appears to be better for nigral hyperintensity assessment than MEDIC acquired with the same resolution or unoptimized whole brain routine clinical SWI and FLAIR sequences. Disease severity may affect the visibility of nigral hyperintensity. Identification of PD based on abnormal nigral hyperintensity appearance may be more reliable in patients with higher MDS-UPDRS II + III composite score and H&Y stage. The disease severity dependent loss of nigral hyperintensity and the promising nature of fast MR imaging techniques should be further investigated in larger samples and in longitudinal follow-up studies of prodromal PD subjects. Combining these future MRI studies with concurrent DaTSCAN imaging may help to answer whether nigrostriatal functional changes or the loss of nigral hyperintensity appear earlier. The promising properties and short measurement time of EPISEG may help the integration of nigral hyperintensity imaging into daily clinical practice.

## Supplementary Information

Supplementary Tables.

## References

[1] Berardelli A (2013). EFNS/MDS-ES/ENS [corrected] recommendations for the diagnosis of Parkinson’s disease. Eur. J. Neurol..

[2] Perlaki G (2016). Validation of an automated morphological MRI-based (123)I-FP-CIT SPECT evaluation method. Parkinsonism Relat. Disord..

[3] Schwarz ST (2014). The ‘swallow tail’ appearance of the healthy nigrosome—a new accurate test of Parkinson’s disease: a case-control and retrospective cross-sectional MRI study at 3T. PLoS ONE.

[4] Schwarz ST (2017). Protocol of a single group prospective observational study on the diagnostic value of 3T susceptibility weighted MRI of nigrosome-1 in patients with parkinsonian symptoms: the N3iPD study (nigrosomal iron imaging in Parkinson's disease). BMJ Open.

[5] Wang Z, Luo XG, Gao C (2016). Utility of susceptibility-weighted imaging in Parkinson’s disease and atypical Parkinsonian disorders. Transl. Neurodegener..

[6] Mahlknecht P, Krismer F, Poewe W, Seppi K (2017). Meta-analysis of dorsolateral nigral hyperintensity on magnetic resonance imaging as a marker for Parkinson’s disease. Mov. Disord..

[7] Kwon DH (2012). Seven-Tesla magnetic resonance images of the substantia nigra in Parkinson disease. Ann. Neurol..

[8] Blazejewska AI (2013). Visualization of nigrosome 1 and its loss in PD: pathoanatomical correlation and in vivo 7 T MRI. Neurology.

[9] Reiter E (2015). Dorsolateral nigral hyperintensity on 3.0T susceptibility-weighted imaging in neurodegenerative Parkinsonism. Mov. Disord..

[10] Noh Y, Sung YH, Lee J, Kim EY (2015). Nigrosome 1 detection at 3T MRI for the diagnosis of early-stage idiopathic Parkinson disease: assessment of diagnostic accuracy and agreement on imaging asymmetry and clinical laterality. AJNR. Am. J. Neuroradiol..

[11] Sung YH, Noh Y, Lee J, Kim EY (2016). Drug-induced Parkinsonism versus idiopathic Parkinson disease: utility of nigrosome 1 with 3-T imaging. Radiology.

[12] Cosottini M (2015). Comparison of 3T and 7T susceptibility-weighted angiography of the substantia nigra in diagnosing Parkinson disease. AJNR. Am. J. Neuroradiol..

[13] Gao P (2016). Universality analysis of the existence of substantia nigra “swallow tail” appearance of non-Parkinson patients in 3T SWI. Eur. Rev. Med. Pharmacol. Sci..

[14] Nam Y, Gho SM, Kim DH, Kim EY, Lee J (2017). Imaging of nigrosome 1 in substantia nigra at 3T using multiecho susceptibility map-weighted imaging (SMWI). J. Magn. Reson. Imaging.

[15] Sung YH (2019). Initial diagnostic workup of parkinsonism: dopamine transporter positron emission tomography versus susceptibility map-weighted imaging at 3T. Parkinsonism Relat. Disord..

[16] Sung YH (2018). Differential involvement of nigral subregions in idiopathic Parkinson’s disease. Hum. Brain Mapp..

[17] Stezin A (2018). Clinical utility of visualisation of nigrosome-1 in patients with Parkinson’s disease. Eur. Radiol..

[18] Sugiyama A (2018). MR findings in the substantia nigra on phase difference enhanced imaging in neurodegenerative parkinsonism. Parkinsonism Relat. Disord..

[19] Kamagata K (2017). Diagnostic imaging of dementia with Lewy bodies by susceptibility-weighted imaging of nigrosomes versus striatal dopamine transporter single-photon emission computed tomography: a retrospective observational study. Neuroradiology.

[20] Bae YJ (2016). Loss of nigral hyperintensity on 3 tesla MRI of Parkinsonism: comparison with (123) I-FP-CIT SPECT. Mov. Disord..

[21] Jin L (2019). Combined visualization of nigrosome-1 and neuromelanin in the substantia nigra using 3T MRI for the differential diagnosis of essential tremor and de novo Parkinson's disease. Front. Neurol..

[22] De Marzi R (2016). Loss of dorsolateral nigral hyperintensity on 3.0 tesla susceptibility-weighted imaging in idiopathic rapid eye movement sleep behavior disorder. Ann. Neurol..

[23] Meijer FJ (2016). Nigrosome-1 on susceptibility weighted imaging to differentiate Parkinson’s disease from atypical parkinsonism: an in vivo and ex vivo pilot study. Pol. J. Radiol..

[24] Gao P (2015). Visualization of nigrosomes-1 in 3T MR susceptibility weighted imaging and its absence in diagnosing Parkinson’s disease. Eur. Rev. Med. Pharmacol. Sci..

[25] Kim EY, Sung YH, Lee J (2019). Nigrosome 1 imaging: technical considerations and clinical applications. Br. J. Radiol..

[26] Calloni SF (2018). Multiparametric MR imaging of Parkinsonisms at 3 tesla: its role in the differentiation of idiopathic Parkinson’s disease versus atypical Parkinsonian disorders. Eur. J. Radiol..

[27] Cheng Z (2020). Imaging the Nigrosome 1 in the substantia nigra using susceptibility weighted imaging and quantitative susceptibility mapping: an application to Parkinson’s disease. Neuroimage Clin..

[28] Oustwani CS (2017). Can loss of the swallow tail sign help distinguish between Parkinson Disease and the Parkinson-Plus syndromes?. Clin. Imaging.

[29] Oh SW (2016). Correlation of 3D FLAIR and dopamine transporter imaging in patients with parkinsonism. AJR. Am. J. Roentgenol..

[30] Berg D (2015). MDS research criteria for prodromal Parkinson’s disease. Mov. Disord..

[31] Makkos A (2018). Are the MDS-UPDRS-based composite scores clinically applicable?. Mov. Disord..

[32] Horvath K (2014). Validation of the Hungarian Mds-Updrs: why do we need a new Parkinson scale?. Ideggyogy. Sz..

[33] Goetz CG, Stebbins GT, Tilley BC (2012). Calibration of unified Parkinson's disease rating scale scores to Movement Disorder Society-unified Parkinson’s disease rating scale scores. Mov. Disord..

[34] Tomlinson CL (2010). Systematic review of levodopa dose equivalency reporting in Parkinson’s disease. Mov. Disord..

[35] Andre JB (2015). Toward quantifying the prevalence, severity, and cost associated with patient motion during clinical MR examinations. J. Am. Coll. Radiol..

[36] Tsai FY, Shih YY, Chan WP, Tsai PH, Chung HW (2012). Practical aspects of shortening acquisition time in brain MR susceptibility-weighted imaging. Neuroradiol. J..

[37] Perez Akly MS (2019). Accuracy of nigrosome-1 detection to discriminate patients with Parkinson’s disease and essential tremor. Neuroradiol. J..

[38] Mandrekar JN (2010). Receiver operating characteristic curve in diagnostic test assessment. J. Thorac. Oncol..

[39] Schwarz ST (2018). Parkinson’s disease related signal change in the nigrosomes 1–5 and the substantia nigra using T2* weighted 7T MRI. Neuroimage Clin..

[40] Horvath K (2017). Minimal clinically important differences for the experiences of daily living parts of movement disorder society-sponsored unified Parkinson’s disease rating scale. Mov. Disord..

[41] Horvath K (2015). Minimal clinically important difference on the Motor Examination part of MDS-UPDRS. Parkinsonism Relat. Disord..

[42] Bae YJ (2018). Loss of substantia nigra hyperintensity at 3.0-T MR imaging in Idiopathic REM sleep behavior disorder: comparison with (123)I-FP-CIT SPECT. Radiology.

[43] Martínez-Martín P (2015). Parkinson’s disease severity levels and MDS-Unified Parkinson’s Disease Rating Scale. Parkinsonism Relat. Disord..

[44] Cosottini M (2014). MR imaging of the substantia nigra at 7 T enables diagnosis of Parkinson disease. Radiology.

[45] Wang N, Yang H, Li C, Fan G, Luo X (2017). Using ‘swallow-tail’ sign and putaminal hypointensity as biomarkers to distinguish multiple system atrophy from idiopathic Parkinson's disease: a susceptibility-weighted imaging study. Eur. Radiol..

[46] Kim JM (2016). Loss of substantia nigra hyperintensity on 7 Tesla MRI of Parkinson's disease, multiple system atrophy, and progressive supranuclear palsy. Parkinsonism Relat. Disord..

