# Supplementary material for: Fast 3 T nigral hyperintensity magnetic resonance imaging in Parkinson’s disease
Source: Sci Rep. 2021 Jan 13;11:1179. doi: 10.1038/s41598-020-80836-7 (PMC7806895; doi:10.1038/s41598-020-80836-7)
Supplement: Supplementary file 1 — Supplementary Tables. [file 41598_2020_80836_MOESM1_ESM.pdf]

# Supplementary Information

## Fast 3 Tesla nigral hyperintensity magnetic resonance imaging in Parkinson's disease

**Gabriella HERNADI MD[1], David PINTER MD PhD[2], Szilvia Anett NAGY PhD[1,3,4,5], Gergely ORSI PhD[1,2,3], Samuel KOMOLY MD PhD[2], Jozsef JANSZKY MD PhD[2,3], Norbert KOVACS MD PhD[2,3], Gabor PERLAKI PhD[1,2,3*]**

[1]Pecs Diagnostic Centre, Pecs, Hungary

[2]Department of Neurology, Medical School, University of Pecs, Pecs, Hungary

[3]MTA-PTE Clinical Neuroscience MR Research Group, Pecs, Hungary

[4]Neurobiology of Stress Research Group, Szentagothai Research Center, University of Pecs, Pecs, Hungary

[5]Department of Laboratory Medicine, Medical School, University of Pecs, Pecs, Hungary

**Running title:** Fast swallow tail imaging

[*]**Corresponding author:**
Gabor PERLAKI PhD
MTA-PTE Clinical Neuroscience MR Research Group
Ret u. 2.
H-7623, Pecs, Hungary
phone: +3672535900 email: petzinger.gabor@gmail.com

# Supplementary Tables

**Supplementary Table S1.** Demographic and clinical data of study participants

| | PD (n=20) | Control (n=25) | P-value |
|---|---|---|---|
| Sex (M/F) | 9/11 | 10/15 | 0.77[a] |
| Age (years) | 59.7±11.4 [42-77] | 63.3±8.0 [43-73] | 0.49[b] |
| Education (years) | 13.5±3.3 [8-21] | 13.0±3.5 [8-21] | 0.69[b] |
| H&Y | stage 1: 9 cases<br>stage 2: 11 cases | n.a. | |
| Disease duration (years) | 3.7±2.1 [1-9] | n.a. | |
| MDS-UPDRS II+III[c] | 16.7±9.6 [2-32]<br>15.0 (9-24.75) | n.a. | |
| MDS-UPDRS II[d] | 5.3±4.8 [0-20]<br>4.0 (2-7) | n.a. | |
| MDS-UPDRS III[d] | 12.4±8.6 [2-28]<br>11.0 (5-22) | n.a. | |
| UPDRS II[d,e] | 4.6±4.3 [0-18]<br>3.0 (2-6) | n.a. | |
| UPDRS III[d,e] | 8.3±7.2 [0-21]<br>7.0 (2-16) | n.a. | |
| LEDD (mg/day) | 200 (100-477.5) | n.a. | |

Data are presented as mean±standard deviation [range]; median (interquartile range).
Abbreviations: PD=Parkinson's disease; M=male; F=female; H&Y=Hoehn-Yahr scale; MDS-UPDRS=Movement Disorder Society-sponsored Unified Parkinson's Disease Rating Scale; MDS-UPDRS II+III=composite MDS-UPDRS Part II and Part III score; UPDRS=Unified Parkinson's Disease Rating Scale; n.a.=not applicable; LEDD=levodopa equivalent daily dose.
[a]Fisher's exact test (2-sided exact P-value)
[b]Mann–Whitney U-test (2-sided exact P-value)
[c]Two patients were excluded (one for missing Part II and one for missing Part III score).
[d]One patient was excluded due to missing score.
[e]Converted from the corresponding MDS-UPDRS score using the formula provided by Goetz et al. [33]

**Supplementary Table S2.** Scan parameters for 3T MRI

| Sequence | TR/TI/TE (ms) | FA (◦) | FOV ($mm^2$) | Matrix Acquired/ reconstructed | $N_{slices}$ | TH (mm) | BW (Hz/px) | TA (min:sec) |
|---|---|---|---|---|---|---|---|---|
| 3D sagittal SPACE FLAIR | 5000/1800/388 | T2 var | 256*256 | 256*256/ 512*512 | 320 | 0.5[a] | 751 | 5:57 |
| 3D axial EPISEG | 150/n.a./36 | 30 | 180*180 | 256*256/ 512*512 | 28 | 1 | 888 | 2:08 |
| 3D axial MEDIC | 64/n.a./35 | 22 | 180*180 | 256*256/ 512*512 | 26 | 1 | 120 | 4:19 |
| 3D axial FLASH | 27/n.a./20 | 15 | 230*172.5 | 256*182/ 256*192 | 88 | 1.5 | 120 | 4:51 |

Abbreviations: TR/TI/TE=repetition/inversion/echo time; FA=flip angle; FOV=field of view; $N_{slices}$=number of slices; TH=slice thickness; BW=bandwidth; TA=time of acquisition; SPACE FLAIR=sampling perfection with application-optimised contrasts using different flip angle evolutions fluid-attenuated inversion recovery; EPISEG=segmented echo-planar imaging; MEDIC=multi echo data image combination gradient echo; FLASH=fast low angle shot; T2 var=variable flip angles across the echo train optimized for T2 contrast; n.a.=not applicable.

[a]nominal slice thickness combined with slice resolution=50%
